# Finger vein recognition based on bilinear fusion of multiscale features

**DOI:** 10.1038/s41598-023-27524-4

**Published:** 2023-01-05

**Authors:** Bin Ma, Kaixuan Wang, Yueli Hu

**Affiliations:** 1 School of Mechatronic Engineering and Automation, Shanghai University, Shanghai, 200444 China; 2 Microelectronics R&D Center, Shanghai University, Shanghai, 200444 China

**Keywords:** Computer science, Image processing, Machine learning

## Abstract

Finger veins are widely used in various fields due to their high safety. Existing finger vein recognition methods have some shortcomings, such as low recognition accuracy and large model size. To address these shortcomings, a multi-scale feature bilinear fusion network (MSFBF-Net) was designed. First, the network model extracts the global features and local detail features of the finger veins and performs linear fusion to obtain second-order features with richer information. Then, the mixed depthwise separable convolution replaces the ordinary convolution, which greatly reduces the computational complexity of the network model. Finally, a multiple attention mechanism (MAM) suitable for finger veins was designed, which can simultaneously extract the channel, spatial, directional, and positional information. The experimental results show that the method is very effective, and the accuracy of the two public finger vein databases is 99.90% and 99.82%, respectively.

## Introduction

With the development of science and technology and the continuous improvement of safety regulations, more and more people pay attention to information security. Biometric identification technology can guarantee people's information security and gradually replace the status of traditional identity identification technology in daily life. Compared with other biometric identification technologies, finger veins have the advantages of high security, privacy, non-contact identification, and low cost due to their hidden under the skin surface. Therefore, finger vein recognition technology has better application prospects and has become a research hotspot in the field of biometric recognition.

Medical research shows that everyone's finger veins are unique, and even twins can't have exactly the same vein texture characteristics^1^. With the increase of age, the pattern of the finger vein will not change. Even if the fingers are worn, the veins of the fingers are not affected. Finger veins can only be collected on a living body using specialized equipment. In recent years, a large number of researchers have carried out research on finger vein recognition technology and have made good progress. Finger vein recognition technology can be divided into two categories: traditional methods of finger vein recognition and deep learning-based finger vein recognition methods^2–4^. The traditional finger vein recognition method preprocesses the finger vein images and then uses traditional algorithms such as PCA, LBP and LDP to extract and classify the texture features of the finger vein images^5,6^. Hu et al. improved the maximum curvature method and proposed that the curvature value of the best direction not only extracted the vein network, but also mined the vein trunk, and verified the effectiveness of the method on two public databases^7^. Yang et al. Proposed a block-based (2D) 2PCA method based on LBP and PCA, which preserves the local information of vein images. The experiment was carried out on FV-USM, and the recognition accuracy was 99.32%^8^. Meng et al. proposed a region-based detail recognition technology to solve the problem of finger vein image distortion. Combining the method based on the region of interest with the method of detail matching, the experiment was carried out on SDUMLA, and excellent results were achieved^9^. Although the traditional methods have a very good performance in the task of finger vein recognition, the recognition results are very dependent on the quality of the image. Before recognition, the finger vein image needs to be preprocessed, and the recognition steps are tedious. With the development of deep learning, more and more neural networks are applied to finger vein recognition tasks. The recognition method based on deep learning has strong feature expression ability. Compared with the traditional finger vein recognition algorithm, it can obtain better results under the same image quality without preprocessing. Kamaruddin et al. proposed a new PCANet to extract semantic information from finger vein images, which has better performance compared with other state-of-the-art methods. This method needs to consider both the original grayscale image and the extracted vein line image^10^. Ren et al. proposed a novel lightweight convolutional neural network that can filter out low-quality images and mine different attributes hidden in low-quality images to improve network performance. The method can be combined with existing databases and recognition algorithms, which guarantees excellent performance of the algorithm, but the recognition time is too long, the recognition time of a single image is 9.2ms^11^. Zhang et al. proposed a joint Bayesian framework based on partial least squares discriminant analysis, which can effectively extract the texture and orientation features of finger veins. Experiments on public databases obtained high recognition accuracy and fast recognition speed. However, when converting the original features into a low-dimensional form, there may be a loss of features^12^. Noh et al. proposed a deep CNN method for finger vein recognition, which fused the texture features and shape features of finger veins, effectively reducing the impact of image noise on the recognition results. This method requires two input images which results in excessive processing time^13^. Hong et al. proposed a novel finger vein recognition network based on VGG16 and achieved excellent results. But the input to the network model needs to use the augmented image, the original unpreprocessed image, and the difference image obtained from the two finger vein images^14^. Yang et al. proposed the FV-GAN network model based on a generative adversarial network, which is the first time to apply a generative adversarial network to the field of finger veins and has strong robustness against outliers and blood vessels ruptures. But requires a large number of training samples^15^. Tang et al. proposed a pre-trained weighted convolutional neural network based on Siamese, which effectively improved the network performance. But the extraction of ROI is not considered^16^. Ren et al. proposed to apply the encryption algorithm to the finger vein recognition task, which greatly improves the security, but due to the need for encryption, it leads to additional time overhead^31^. It can be seen from the above analysis that the existing identification methods have achieved good results in recognition performance, but there are still some shortcomings in recognition time and computational complexity. The method in this paper comprehensively considers the four aspects of recognition accuracy, model parameters, computational complexity, and recognition time, and obtains good performance.

The methods based on deep learning have achieved better results without image preprocessing. However, the quality of the image is still an important factor affecting the performance of the network model. When the image quality is too poor or the database samples are too few, it is difficult for the network to extract the effective features of finger veins, so there is a disadvantage that the recognition accuracy is not high enough. Convolutional neural networks also have shortcomings such as many model parameters, and long training and recognition time. To solve these problems, we propose a multi-scale feature bilinearly fusion network combined with the multiple attention mechanism. Firstly, a multi-scale feature bilinear fusion network is designed. The shallow structure of the network model extracts the detailed features of finger veins, and the deep structure extracts the overall contour of finger veins. Then the shallow features and deep features are bilinear fused to obtain the second-order features with richer information. Secondly, the lightweight method based on mixed depthwise separable convolution is adopted to reduce the parameters and computational complexity of the network, and speed up network model training and recognition. Finally, to further improve the recognition accuracy, a multiple attention mechanism module is designed and embedded into the network model to simultaneously extract the space, channel, direction, and position information of the finger vein images .

## Proposed method

In this section, a novel end-to-end finger vein recognition network framework is proposed to extract finger vein features.

### Multi-scale feature bilinear fusion network

**Figure 1:**
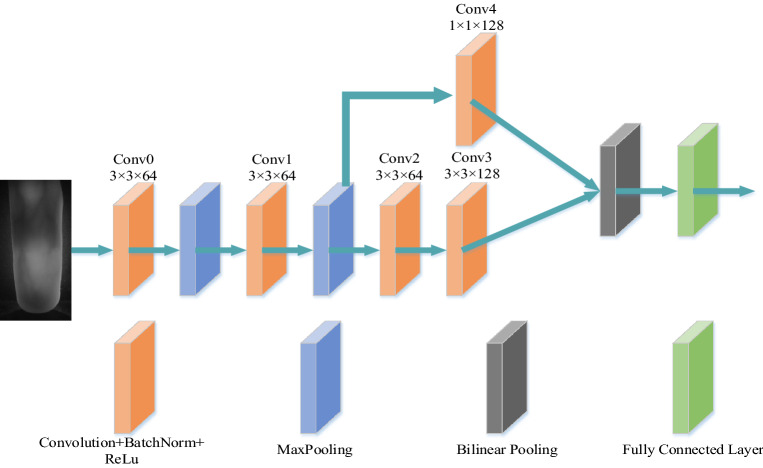
Multi-scale feature bilinear fusion network structure.

Finger veins are composed of vein patterns and have relatively simple features, which results in a certain similarity between different finger veins. Recognition only by extracting vein pattern features will leads to recognition errors. To solve these problems, a multi-scale feature bilinear fusion network was designed. The network structure is shown in Fig. 1.

Take the Conv0 layer as an example, where 3 × 3 represents the size of the convolution kernel, and 64 represents the number of convolution kernels. Firstly, the shallow features of the finger vein image are extracted by two convolution layers. The shallow features include the grain details of the finger vein, and the shallow features are divided into two paths, one of which is reserved for classification. The other way continues to use two convolution layers to extract the deep features including outline of the finger vein from the shallow features. The retained shallow features are dimensionally increased by using a 1 × 1 convolution kernel, and bilinear fusion is performed with deep features to obtain second-order features containing rich information. The formula is as follows:

1 $$ b(p,I,f_{1} ,f_{2} ) = f_{1}^{T} (p,i)f_{2} (p,i) $$

where *f*_1_(*p*,*i*) ∈ R^*T*×*M*^ and *f*_2_(*p*,*i*) ∈ R^*T*×*M*^ is the deep feature and shallow feature of image *i* at position *p*. The deep feature and shallow feature are bilinearly fused to obtain matrix *b*, which contains the correlation of these two features.

2 $$ z = vec(\sum\limits_{p} {b(p,i,f_{1} ,f_{2} )} ) $$

Equation (2) uses sum pooling to aggregate bilinear features on *b* of all positions in image *i*, and the feature dimension is *M* × *M*. In the convolutional neural network, the fully connected layer is responsible for classifying the final features, while the fully connected layer can only accept one-dimensional feature vectors, so the features need to be flattened to obtain *z* ∈ R^(*M*×*M*)×1^.

3 $$ x = sign(z)\sqrt {\left| z \right|} $$

4 $$ y = \frac{x}{{\left\| x \right\|_{2} }} $$

Equation (3) normalizes the obtained *z* vector. Equation (4) is to perform L2 normalization on the vector *x*, where ||*x*||_2_ represents the norm of the vector *x*. The signed square root and L2 normalization operations are performed on the obtained one-dimensional features, which can improve the operation speed of the fully connected layer, avoid overfitting, and increase the network performance. Finally, the fully connected layer processes the feature *y* to complete the classification of finger vein images.

Since the finger vein features are relatively simple, the network model is not very deep, resulting in high efficiency. The multi-scale feature bilinear fusion network is divided into two branches. One branch locates the finger veins and extracts the shallow features (local features) of the finger veins. The other branch continues to process the shallow features and extracts the deep features (vein contours). The two-way features are bilinear fused to obtain second-order features, which contain more useful information. The global and local features of finger vein are fully mined, which makes the prediction and classification of finger vein recognition task more accurate.

### Network lightweight

The convolutional neural network has a large number of parameters and computations, which ensures the powerful visual feature expression ability of the network model, but pays the cost of parameter redundancy, which will reduce the training and recognition speed. Because the finger vein recognition algorithm must operate in real-time on hardware with constrained resources, there are somewhat strict limitations on the size of the network model and the number of model parameters. It is required to create a lightweight network model in order to condense the model's size and satisfy the neural network's low-latency operating criteria. We adopted a shallower bilinear fusion network with deep multiscale features, allowing for a smaller number of parameters in the fully connected layer. The lightweight method in this paper is to directly optimize the convolutional layer and replace the conventional convolution with a mixed depthwise separable convolution to reduce the computational complexity of the network model.

The depthwise separable convolution decomposes the convolution process and divides the ordinary convolution into depthwise convolution and pointwise convolution, which greatly reduces the number of parameters and computations. Generally, the depthwise separable convolution uses a 3 × 3 convolution kernel, which is not suitable for finger vein recognition tasks. Due to the particularity of the structure of finger veins, the combination of large receptive field features and small receptive field features can obtain better results. The mixed depthwise separable convolution divides the feature map channels into groups, and each group uses depthwise separable convolutions with different kernel sizes, which can extract receptive field features of different sizes, so it is more suitable for finger vein recognition tasks.

**Figure 2:**
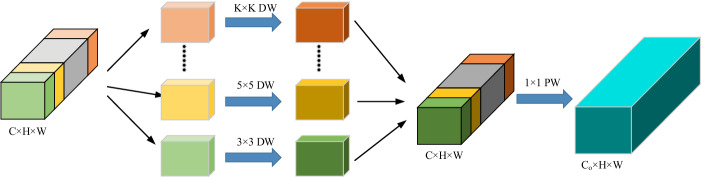
Mixed depthwise separable convolution.

Figure 2 shows the convolution process of the mixed depthwise separable convolution. As can be seen from the figure, the mixed depthwise separable convolution receives input tensors, groups the channels of the tensors, and each group uses convolution kernels of different sizes. The features of different receptive fields can be extracted in the same convolution layer, and then different groups of features are combined together. The depthwise separable convolution is divided into two steps, which just separate feature extraction and feature combination. The tensors output in the feature extraction stage will be discharged according to the size of the receptive field, and the output tensors will be mixed in the feature combination stage, so there is no need to worry about the transmission of different receptive fields characteristics. The multi-scale feature bilinear network extracts the shallow features and deep features of the finger vein images. Therefore, the lightweight idea is to use a single set of 3 × 3 mixed depth separable convolutions in the shallow network to extract the contour features of the finger veins, and use 3 × 3 and 5 × 5 mixed depthwise separable convolutions in the deep network to extract the detailed features of different receptive fields of finger vein. To further reduce the computational complexity of the network model, the 5 × 5 convolution kernel is replaced with a 3 × 3 (dilation = 2) dilated convolution. The network structure is shown in Table 1.

**Table 1:** Multi-scale feature bilinear fusion network lightweight structure.

Layer number	Layer name	Kernel number	Kernel size
Conv0	Mixed depthwise separable convolution	64	[3]
Max0	Max pooling	64	2
Conv1	Mixed depthwise separable convolution	64	[3]
Max1	Max pooling	64	2
Conv2	Mixed depthwise separable convolution	64	[3,3(dilation = 2)]
Conv3	Mixed depthwise separable convolution	128	[3,3(dilation = 2)]
Conv4	Mixed depthwise separable convolution	128	[1]

Compared with ordinary convolution, the mixed depthwise separable convolution has significantly reduced the computational cost and the number of parameters, resulting in a better performance in terms of lightweight. However, depthwise separable convolution also has some shortcomings that cannot be ignored. The depthwise convolution performs independent convolution on each channel of the input tensor, ignoring the spatial location information. Pointwise convolution performs a weighted combination of input tensors in depth, which does not make efficient use of channel information. The combination of them separates the channel and space of the convolution, which affects the transfer of feature information. In response to this problem, a multiple attention mechanism module suitable for finger veins is designed, which will be described in detail in the next section.

### Multiple attention mechanism

**Figure 3:**
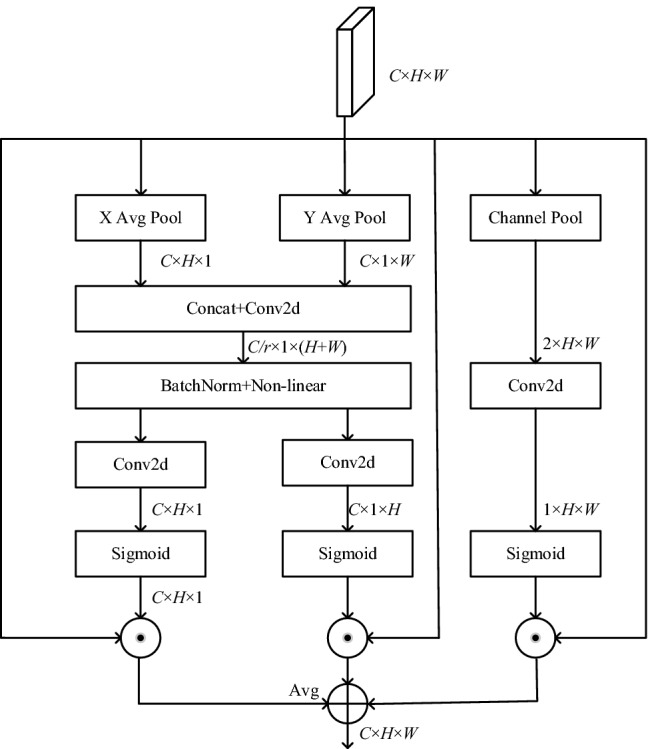
Multiple attention mechanism.

For the finger vein recognition task, a network that can extract key features is very important. However, only relying on the basic network to extract finger vein features for prediction and classification, the results often fail to meet their expectations. Firstly, finger vein recognition demands high quality images, while collecting clear and noiseless high-quality finger vein image is always challenging. Secondly, A convolutional neural network will not locate the region of interest of the finger vein map like the human brain but process the whole image, which will extract many useless features. The process of feature classification is a waste of time and will be misled by these useless features, leading to classification errors. To overcome above two issues, The attention mechanism is adopted to save the energy wasted on useless information, and invest more in the key area in order to obtain detailed information in the area. Existing attention mechanisms are roughly divided into spatial attention mechanisms and channel attention mechanisms. If you want to use these two modules at the same time, it is also realized through a series connection, and the information may be lost in the transmission between modules. In view of the characteristics of finger veins and the shortcomings caused by lightweight processing, a multiple receptive field module is designed. The structure is shown in Fig. 3.

The multiple attention mechanism receives an input tensor *X* ∈ R^*C*×*H*×*W*^ and outputs a tensor *Y* ∈ R^*C*×*H*×*W*^ with feature enhancement capability. The multiple attention mechanism module is divided into multiple branches for parallel processing. First, the input tensor enters the module, and the first two branches are pooled by (*H*, 1) and (1, *W*) through X Avg Pool and Y Avg Pool respectively. Each channel encoding is checked so that features are aggregated along two spatial directions, capturing spatial interactions with location information. The output of channel *c* with height h and width w can be obtained as follows:

5 $$ s_{c}^{h} = \frac{1}{W}\sum\limits_{1 \le i \le H} X $$

6 $$ s_{c}^{w} = \frac{1}{H}\sum\limits_{1 \le i \le W} X $$

After helping the network model to find the region of interest of the finger vein, the two-way features are cascaded, and then the 1 × 1 size convolution is used to reduce the dimension of the features, which reduces the computational complexity. The formula is as follows:

7 $$ f = \delta (F_{1} ([s^{h} ,s^{w} ])) $$

where *F*_1_ represents a 1 × 1 size convolution operation, and δ represents non-linear activation function. Finally, *f* is divided into two independent tensors along the spatial dimension, and the sigmoid function is used to limit the features to 0–1 after using 1 × 1 convolution to increase the dimension. The final result is used as a weight parameter to enhance important features and weaken unimportant feature information:

8 $$ g^{h} = \sigma (F_{1} (f^{h} )) $$

9 $$ g^{w} = \sigma (F_{1} (f^{w} )) $$

The first two branches complete the horizontal and vertical directions of channel attention and then introduce the spatial attention branch. After the input feature map is pooled, the channel dimension becomes 1, and the spatial information is aggregated to obtain average pooling feature *F*_avg_ ∈ R^1×*H*×*W*^ and max pooling features *F*_max_ ∈ R^1×*H*×*W*^ respectively. The two feature maps are then concatenated together, and a conventional convolution is used to generate an attention image with spatial information. The calculation method is as follows:

10 $$ \begin{gathered} g = \sigma \left( {F_{7} ([AvgPool(X);MaxPool(X)])} \right) \\ = \sigma (F_{7} ([f_{avg}^{S} ;f_{\max }^{S} ])) \\ \end{gathered} $$

where *σ* represents the sigmoid function and *F*_7_ represents a 7 × 7 size convolution operation. The sigmoid function can assign corresponding weights according to the different degrees of information contribution at different positions. Equation (11) multiplies and sums multiple input features and the weights obtained by the corresponding attention mechanism, and then calculates the average value to obtain the final feature

11 $$ Y = \frac{{(X \times g^{h} + X \times g^{w} + X \times g)}}{3} $$

**Figure 4:**
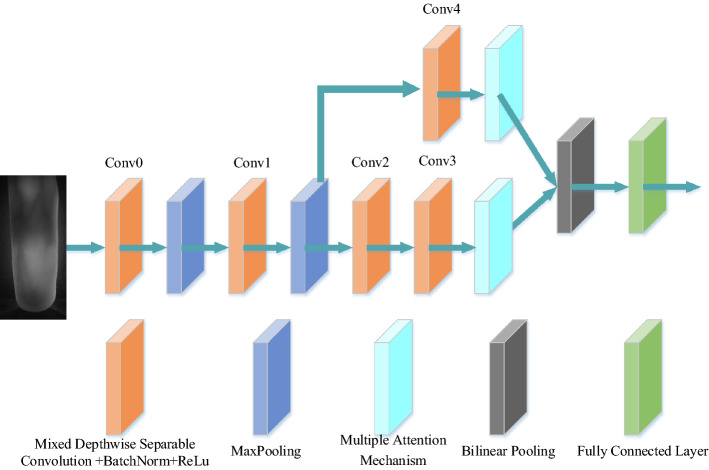
Proposed lightweight MSFBF-Net with attention mechanism.

The multiple attention mechanism embeds the position information into the channel attention and combines with the spatial attention mechanism, which not only extracts the channel domain and spatial domain information, but also captures the directional and positional information of the finger veins, and performs attention operations in large areas. Improved network performance at a lower computational cost. The multiple attention mechanism assigns weights to different information on the finger vein image according to the degree of contribution, which is very suitable for finger vein recognition tasks and makes up for the deficiencies caused by depthwise separable convolution. The combination of a multi-scale feature bilinear fusion network with mixed depthwise separable convolution and multiple attention mechanism is the final model proposed for the finger vein recognition task in this paper. The network structure is shown in Fig. 4.

## Experiments and analysis

### Finger vein database

To evaluate the performance of the proposed network model from multiple aspects, we conduct experiments on two public finger vein databases, SDUMLA^17^ and FV-USM^18^.

#### SDUMLA

SDUMLA considers the influence of translation, rotation, and other factors during acquisition, so the image quality is slightly worse. A total of 106 volunteers participated in the collection work. The index finger, middle finger, and ring finger of both hands were collected. Each finger captured six images. Finally, 636 finger vein categories and 3816 finger vein images were obtained, and the image size was 320 × 240.

#### FV-USM

The quality of vein image collection in FV-USM is high. 123 volunteers participated in the collection work. Six images of their left and right index fingers and middle fingers were collected respectively. The images were collected in two times, and six finger vein images were captured in each collection. Finally, 492 finger vein categories and 5904 finger vein images are obtained, and the image size is 640 × 480.

The public finger vein database takes into account differences in age, gender, and region of volunteers when collecting, and also considers the influence of non-ideal images such as angle, occlusion, and dark light. In order to facilitate the analysis and comparison of the experimental results, the database image size is adjusted to 60 × 180. The pictures in the public database are randomly divided according to the ratio of 1:5, one is the test database, and the five are the training database, to ensure that they are independent of each other.

### Evaluation indicators

This paper uses the recognition accuracy, recognition time, Parameter and Flops as the evaluation indicators of the model. The recognition accuracy represents the number of correct classifications by the network model, and the recognition time is the time it takes the network to recognize a picture. Parameter(M) stands for Mega-parameter, Flops(GFlops) stands for Giga float point operations, which can be used as a measure of whether it is suitable for hardware deployment.

### Training configuration

We implement our proposed method using PyTorch and trained the model on the NVIDIA GeForce GTX1660 (6 GB) GPU. Adam optimizer is employed during training, and the batch size is set at 8. We trained the model using dataset FV-USM and SDUMLA respectively. When trained on dataset FV-USM, the duration of a training epoch is 5.86 s. The final trained model is obtained after 300 epochs. Thus, the total training time is 1758s. When trained on dataset SDUMLA, Each epoch takes 4.06 s to train. The final trained model is also obtained after 300 epochs. Thus the total training time is 1218 s.

### Comparison with classical network

As can be seen from the data from Table 2, compared with the classical network model, the multi-scale feature bilinear fusion network performs very well. This paper proposes MSFBF-Net, and then obtains two variant networks based on this network model, which are the lightweight MSFBF-Net(Lightweight MSFBF-Net) and the lightweight MSFBF-Net combined with multiple attention mechanism(Lightweight MSFBF-Net + MAM). The recognition accuracy of MSFBF-Net on two public databases is as high as 99.59% and 99.53%. Single image recognition time is only about 3 ms. Both model parameters and computational complexity have certain advantages. After a comprehensive comparison, it is concluded that the multi-scale feature bilinear fusion network is the most suitable for the finger vein recognition task.

**Table 2:** Comparison of classic network performance.

Method	Accuracy/%		Time/ms	Parameter/M	Flops/GFlops
	FV-USM	SDUMLA			
VGG-16^19^	95.83	95.71	49	44.0	3.20
DesNet^20^	96.95	98.16	34	26.7	0.17
AlexNet^21^	92.28	96.32	16	19.7	0.43
ResNet-18^22^	96.03	97.79	16	11.0	2.40
MSFBF-Net	99.59	99.53	3	8.2	0.21

### Ablation experiment

In order to verify the correctness of ideas and methods and the effectiveness of each step, it is necessary to conduct ablation experiments. The algorithm is evaluated from the following four aspects: recognition accuracy, model parameters, computational complexity, and single image recognition time. Next, FV-USM data are used for analysis, and the experimental results are shown in Table 3. Although the multi-scale feature bilinear fusion network model has obtained a good recognition accuracy, the parameters and computational complexity are very large. After the network model is lightweight, a lot of computational complexity is reduced, and the recognition accuracy decreased from 99.59 to 99.29%, which is consistent with our expectation. The decrease in recognition accuracy is due to the use of mixed depthwise separable convolutions in the network. The depthwise separable convolution destroys the correlation between channels and spaces, resulting in a decrease in recognition accuracy. Finally, the multiple attention mechanism is embedded into the lightweight network model. It can be seen that the accuracy rate has increased from 99.29% to 99.90%, and the amount of computation has hardly increased, still 0.043GFlops. It is very convenient for the model to be deployed on hardware devices later. Comparing the lightweight model with the novel lightweight network model, we find that the method in this paper maintains high recognition accuracy, low computational complexity and less recognition time, while the amount of model parameters is acceptable. This is because the model parameters are composed of convolution kernel parameters and weight parameters. There are few convolution layers in our network model, so it is not obvious to reduce the parameters of the lightweight model. But most of the computation is in convolutional layers, which reduces Flops drastically from 0.21 to 0.043 GFlops. The recognition time of a single image of the final model is only 4 ms. This is consistent with the theoretically expected results, demonstrating the effectiveness of the algorithm at each step.

**Table 3:** Ablation experiment results.

Method	Accuracy/%		Time/ms	Parameter/M	Flops/GFlops
	FV-USM	SDUMLA			
EfficientNet-B0^23^	99.09	98.58	12	5.3	0.069
MobileNet-V1^24^	94.72	94.18	4	3.7	0.14
GhostNet^25^	95.83	95.60	11	4.7	0.039
MSFBF-Net	99.59	99.53	3	8.2	0.21
Lightweight MSFBF-Net	99.29	99.21	2	8.1	0.043
Lightweight MSFBF-Net + MAM	99.90	99.82	4	8.1	0.043

### Compared with the traditional algorithm

Table 4 shows the experimental results on FV-USM. It can be seen that the recognition accuracy of the lightweight multi-scale feature bilinear fusion network combined with multiple attention mechanism is better than that of the excellent traditional finger vein recognition algorithm. In addition, when the network model is used for finger vein recognition, the finger vein database image is directly used without preprocessing, which greatly reduces the time cost.

**Table 4:** Experimental results of traditional algorithms.

Method	Accuracy/%
LBP	96.10
LBP + SVM	97.86
SURF^26^	97.17
Yang et al.’s method^27^	99.52
Ours	99.90

### Compared with existing deep learning methods

**Figure 5:**
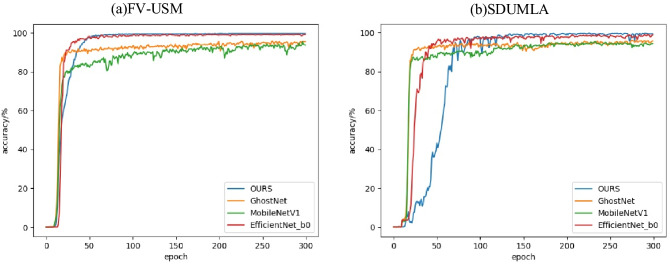
Recognition accuracy curve.

**Figure 6:**
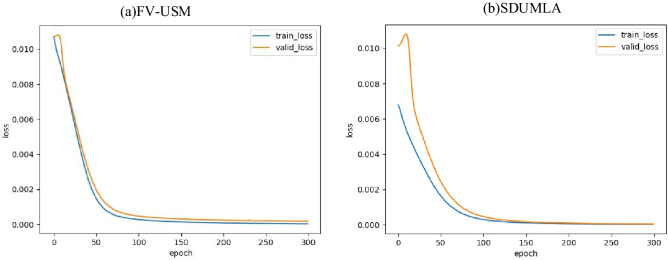
Loss curve.

The lightweight multi-scale feature bilinear fusion network combined with the multiple attention mechanism and the existing convolutional neural network are tested on two public databases, and the experimental results are shown in the Table 5. Among the excellent finger vein recognition algorithms in recent years, Ren et al.'s method has the best comprehensive performance, but our method has better performance in recognition accuracy and recognition time. Figure 5 shows the recognition curves of our method and some other network models on two public databases. It can be seen that our method has advantages in recognition accuracy. Figure 6 is the training loss curve and valid loss curve obtained by using the lightweight MSFBF-Net architecture with attention mechanism proposed in this paper to experiment with FV-USM and SDUMLA. It can be seen from the figure that the model gradually converges after 100 epochs.

**Table 5:** Experimental results of deep learning models.

Method	Accuracy/%		Time/ms
	FV-USM	SDUMLA	
Zhao et al.’s method^28^	97.95	98.41	9
Shen et al.’s method^29^	99.59	99.30	14
Shaheed et al.’s method^30^	99.08	98.50	45
Ren et al.’s method^31^	99.59	96.70	5
Ours	99.90	99.82	4

**Figure 7:**
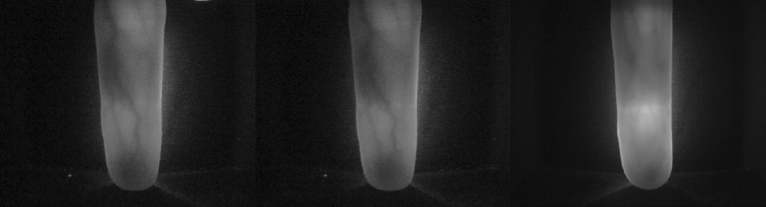
Poor quality images in public databases.

Figure 7 shows some poor-quality images with obvious noise and unclear vein lines in public databases. Other methods of identification always identify errors. The lightweight multi-scale feature bilinear fusion network combined with multiple attention mechanism is designed for the characteristics of finger veins, which can extract the multi-scale features with rich information, and simultaneously extract the channel, spatial, directional, and positional information to assist recognition.

## Conclusion

Aiming at the problems of large models, many parameters, and long recognition time in existing finger vein recognition methods, a lightweight multi-scale feature bilinear fusion network combined with multiple attention mechanism was proposed. Firstly, the multi-scale feature bilinear fusion network extracts the global features and local detail features of the finger vein image. After bilinear pooling, a second-order feature containing richer information is obtained. Then, the mixed depthwise separable convolution is used to lightweight the network, which greatly reduces the computational complexity of the network. Finally, a multiple attention mechanism is designed to make up for the shortcomings of the mixed depthwise separable convolution and strengthen the network model's ability to extract subtle features. The proposed method is tested on two public databases, and the recognition accuracy is up to 99.90% and 99.82%, which is 7.62% higher than other methods. After the lightweight processing of the network, the computational complexity is only one-fifth of the original, and the recognition time of a single image is only 4 ms. Comprehensive comparison, the effect of the method in this paper is significantly improved, which is very suitable for finger vein recognition tasks.

## Data Availability

The data that support the findings of this study are available from SDUMLA (https://time.sdu.edu.cn/kycg/gksjk.htm) and FV-USM (http://drfendi.com/fv_usm_database/) but restrictions apply to the availability of these data, which were used under license for the current study, and so are not publicly available. Data are however available from the corresponding author upon reasonable request and with permission of official.
